# Keratin 19 as a key molecule in progression of human hepatocellular carcinomas through invasion and angiogenesis

**DOI:** 10.1186/s12885-016-2949-y

**Published:** 2016-11-18

**Authors:** Masato Takano, Keiji Shimada, Tomomi Fujii, Kohei Morita, Maiko Takeda, Yoshiyuki Nakajima, Akitaka Nonomura, Noboru Konishi, Chiho Obayashi

**Affiliations:** 1 Departments of Diagnostic Pathology, Nara Medical University School of Medicine, 840 Shijo-cho, Kashihara, Nara 634-8521 Japan; 2 Department of Pathology, Nara Medical University School of Medicine, 840 Shijo-cho, Kashihara, Nara 634-8521 Japan; 3 Department of Surgery, Nara Medical University School of Medicine, 840 Shijo-cho, Kashihara, Nara 634-8521 Japan; 4 Hokuriku CPL, 15-36 Ninomiya-cho, Kanazawa, Ishikawa 920-0067 Japan

**Keywords:** Keratin 19, Hepatocellular carcinoma, Senescence, Apoptosis, Angiogenesis

## Abstract

**Background:**

Keratin (K) 19-positive hepatocellular carcinoma (HCC) is well known to have a higher malignant potential than K19-negative HCC: However, the molecular mechanisms involved in K19-mediated progression of HCC remain unclear. We attempted to clarify whether K19 directly affects cell survival and invasiveness in association with cellular senescence or epithelial-mesenchymal transition (EMT) in K19-positive HCC.

**Methods:**

K19 expression was analysed in 136 HCC surgical specimens. The relationship of K19 with clinicopathological factors and survival was analysed. Further, the effect of K19 on cell proliferation, invasion, and angiogenesis was examined by silencing *K19* in the human HCC cell lines, HepG2, HuH-7, and PLC/PRF/5. Finally, we investigated HCC invasion, proliferation, and angiogenesis using K19-positive HCC specimens.

**Results:**

Analysis of HCC surgical specimens revealed that K19-positive HCC exhibited higher invasiveness, metastatic potential, and poorer prognosis. In vitro experiments using the human HCC cell lines revealed that *K19* silencing suppressed cell growth by inducting apoptosis or upregulating *p16* and *p27*, resulting in cellular senescence. In addition, transfection with *K19* siRNA upregulated E-cadherin gene expression, significantly inhibited the invasive capacity of the cells, downregulated angiogenesis-related molecules such as *vasohibin-1* (*VASH1*) and *fibroblast growth factor 1* (*FGFR1*), and upregulated *vasohibin-2* (*VASH2*). K19-positive HCC specimens exhibited a high MIB-1 labelling index, decreased E-cadherin expression, and high microvessel density around cancer foci.

**Conclusion:**

K19 directly promotes cancer cell survival, invasion, and angiogenesis, resulting in HCC progression and poor clinical outcome. K19 may therefore be a novel drug target for the treatment of K19-positive HCC.

**Electronic supplementary material:**

The online version of this article (doi:10.1186/s12885-016-2949-y) contains supplementary material, which is available to authorized users.

## Background

Liver cancer is the second leading cause of cancer death in men worldwide. In 2012, the incidence of liver cancer was estimated at 782,500 and 745,500 deaths were associated with this disease [1]. Primary liver cancers have traditionally been classified into hepatocellular carcinoma (HCC) and cholangiocellular carcinoma (CCC) originating from hepatocytes and cholangiocytes, respectively [2]. In normal human liver, hepatocytes typically express keratin (K) 8 and K18, while bile duct cells predominantly express K7 and K19 [3]. In previous studies, a subset of HCC was observed to express K19 [3–10]. Durnez et al. [4] showed that K19-positive HCC cells were characterized by an oval nucleus and a narrow rim of cytoplasm, resembling non-neoplastic hepatic progenitor cells. Given this phenotype, these researchers hypothesized that these cells may be derived from progenitor cells that have the bipotential to differentiate into both hepatocytes and cholangiocytes. Interestingly, K19-positive HCC had a significantly higher incidence of early recurrence and metastasis to extrahepatic organs, including regional lymph nodes, compared to K19-negative (conventional) HCC [5]. Aggressive clinical behavior and poor prognosis of K19-positive HCC are thought to be due to frequent vascular invasion, poor differentiation, or high proliferative activity of these cells, as identified by immunohistochemical assessment of Ki-67 [3, 6, 8]. Several studies using a tissue microarray or snap-frozen human HCC tissue samples demonstrated that both protein and mRNA levels of the molecules associated with epithelial-mesenchymal transition (EMT), such as vimentin, S100A4, and snail, were highly elevated, but decreased expression of E-cadherin was observed less frequently in K19-positive HCC [8].

The mechanisms responsible for the increased malignancy of K19-positive HCC compared to conventional K19-negative HCC have been previously explored in the study by Govaere et al. [11]. In the present study, we attempted to clarify whether K19 affects cell survival and invasiveness directly in association with cellular senescence or EMT in K19-positive HCC.

## Methods

### Patients and tissue specimens

Tissue specimens were collected from 136 patients with HCC who underwent primary curative hepatectomy at the Nara Medical University Hospital, during the period between 2007 and 2012. No other treatments were given before resection. There were 103 men and 33 women with an age range of 29 to 84 (mean 69) years. Of the 136 HCC cases, 33 (24.3%) were positive for hepatitis B virus surface antigen (HBsAg), 62 (45.6%) were positive for hepatitis C virus antibody (HCVAb), and 43 (31.6%) were negative for both HBsAg and HCVAb. The follow-up period from surgical treatment until death due to HCC (16 cases) or the end of this study was 30 to 2550 days (mean 1100 days).

Tissues were fixed in 10% formalin, embedded in paraffin, cut into 3 μm sections, and mounted on silane-coated slides. One section from each tissue was stained with hematoxylin and eosin for histological examination. The diagnosis of HCC was based on WHO criteria [2]. Recurrence was diagnosed by biochemical tests (tumour marker; Alpha-fetoprotein, protein induced by vitamin K absence or antagonist-II), sonograms, computed tomography (CT) and magnetic resonance imaging (MRI). Written informed consent was obtained from all patients before treatment, according to our institutional guidelines. This study was approved by the institutional review board.

### Immunohistochemistry

Immunohistochemical study was performed on paraffin sections using a BOND MAX Automated Immunohistochemistry Vision Biosystem (Leica Microsystems, Wetzlar, Germany). For antigen retrieval step, Bond Epitope Retrieval Solution 1 (citrate-based solution, pH 6.0) (Leica Biosystems, Nussloch, Germany) was used. Antibodies for immunohistochemistry are listed in Table 1. Double immunostaining was carried out following manufacturer’s protocols using K19 and the Bond Polymer Refine Detection kit (brownish colour, Leica Biosystems), and E-cadherin and the Bond Polymer Refine AP-Red Detection kit (red colour, Leica Biosystems). Bile ducts, liver, lymph nodes, vascular endothelium, and endothelial layer of the human placenta were used as positive control for K19, E-cadherin, Ki-67, CD31, and VASH1, respectively. Negative controls were carried out by substitution of the primary antibodies with non-immunized mouse serum, resulted in no signal detection (Additional file 1: Fig. S1). In this study, K19-positive HCC was defined as that in which > 5% of total carcinoma cells showed immunoreactivity against K19. E-cadherin, Ki-67, and VASH1 positive cells were counted in 1000 cancer cells from K19-positive and K19-negative areas in K19-positive HCC specimens. The number of blood vessels in K19-positive and K19-negative HCC specimens, identified by CD31 around cancer foci, was counted in 10 high-power fields (100×.).

**Table 1:** List of antibodies for immunohistochemistry

Primary antibody	Clone	Species	Source	Dilution	Staining reagent
K19	B170	Mouse	Leica Biosystems, Nussloch, Germany	1:300	DAB
E-cadherin	36B5	Mouse	Leica Biosystems	1:50	AP
Ki-67	MIB-1	Mouse	Life Technologies, Carlsbad, CA, USA	Predilution	DAB
CD31	JC70A	Mouse	DAKO, Glostrup, Denmark	1:200	DAB
VASH1	4A3	Mouse	Abnova, Taipei, Taiwan	1:1500	DAB

*Abbreviations*: *DAB* diaminobenzidene, *AP* alkaline phosphatase

### Cell culture

The human HCC cell lines, HepG2, HuH-7, and PLC/PRF/5 were purchased from Japanese Collection of Research Bioresources Cell Bank (Osaka, Japan) and cultured in RPMI supplemented with 10% FBS.

### Transfection of human *K19* siRNA in vitro

The cells were seeded at 10^5^ cells per well in 6-cm plates, and transfected with 100 nmol/L control RNA (Santa Cruz bio, Dallas, TX, USA) or human *K19* siRNAs using Lipofectamine RNAiMAX (Life Technologies, Carlsbad, CA, USA), in accordance with the manufacturer's protocol. After culturing for the indicated time, the samples were removed and homogenized.

### Quantitative real-time PCR

Template cDNA was synthesised from 1 μg of total RNA using Primer Script RT reagent Kit (Takara, Shiga, Japan). The quantitative real-time PCR detection was performed using a SYBR® Premix Ex Taq kit (Takara). The amount of actin mRNA in each sample was used to standardise the quantity of each mRNA. The sequences of the primers used for PCR are shown in Table 2.

**Table 2:** The sequences of the primers for PCR used in this study

Gene	Sequences (5’-3’)
Actin	ATGGGTCAGAAGGATTCCTATGT
	GAAGGTCTCAAACATGATCTGGG
K19	TACAGCCACTACTACACGACCATC
	AGAGCCTGTTCCGTCTCAAACT
E-cadherin	CAGCGTGTGTGACTGTGAAGG
	CAGCAAGAGCAGCAGAATCAGAA
vimentin	TGGCCGACGCCATCAACACC
	CACCTCGACGCGGGCTTTGT
p16	GCTTCCTGGACACGCTGGT
	CGGGCATGGTTACTGCCTCTG
p27	CCGGCTAACTCTGAGGACAC
	TTGCAGGTCGCTTCCTTATT
N-cadherin	ACGCCGAGCCCCAGTATC
	GGTCATTGTCAGCCGCTTTAAG
snail	CCTGCGTCTGCGGAACCT
	TTGGAGCGGTCAGCGAAGG
vasohibin-1 (VASH1)	ACATGCGGCTCAAGATTGGC
	TCACCCGAGGGCCGTCTT
vasohibin-2 (VASH2)	CAGGGACATGAGAATGAAGATCCT
	CAGGCAGTGCAGGCGACT
FGFR1	GCCTGAACAAGATGCTCTCC
	CAATATGGAGCTACGGGCAT

*Abbreviations*: *K* keratin, *FGFR* fibroblast growth factor

### Cell proliferation assay 

For the cell proliferation assay, the methane thiosulfonate (MTS) reagent was used as previously described [12–14]. All the experiments were performed in triplicate.

### Cell invasion assay

In vitro invasion assays were performed using Matrigel invasion chambers (BD Biosciences, Bedford, MA, USA) as previously described [15]. Invading cells were counted under a light microscope. The experiment was repeated three times.

### Senescence assay

Cells were fixed at 70% confluence and then incubated at 37 °C overnight with staining solution containing X-gal substrate (Senescence Detection kit, BioVision, Milpitas, CA, USA). Cells were then observed under a microscope for the presence of blue stain [16].

### Detection of apoptosis

Liquid based cytology (LBC) was used to prepare the cell lines for apoptosis assay by terminal deoxynucleotidyl transferase-mediated deoxyuridine triphosphate-biotin nick end labelling (TUNEL) using the ApopTag in situ apoptosis detection kit (Oncor, Gaithersburg, MD, USA) [17]. We identified cells showing darkly stained nuclei or nuclear fragments as TUNEL-positive apoptotic cells, and counted those in several high-power fields.

### Statistical analysis

Differences in continuous variables were analysed using ANOVA or nonparametric tests (Mann–Whitney and Kruskal–Wallis tests). All the experimental results were analysed using the 1-way analysis of variance and Tukey’s post-hoc test. The 2-tailed student’s t-test was used to compare 2 data points. The survival curves were calculated by the Kaplan-Meier method, and the differences between curves were analysed by the log-rank test. Multivariate analysis for overall survival was performed using a Cox regression model with forward stepwise selection. The results were considered to be statistically significant if *p* < 0.05.

## Results

### Clinicopathological features and prognosis of K19-positive HCC

**Fig. 1:**
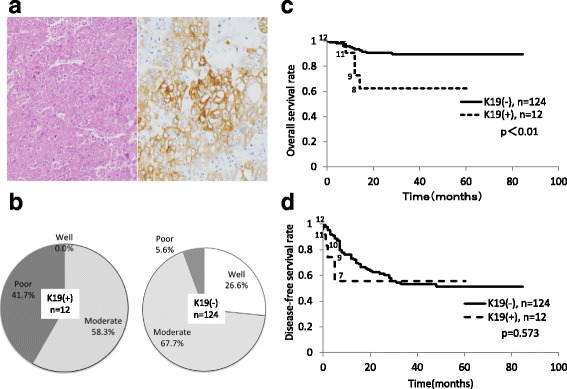
Clinicopathological features of K19-positive HCC. **a** A sample of K19-positive HCC specimen stained with hematoxylin and eosin (HE) and K19 immunostaining. Original magnifications: ×100 (HE), ×200 (CK19). **b** Poorly differentiated HCC more frequently expressed K19. **c** Overall survival rate and **d** disease-free survival rate after primary curative hepatectomy of HCC patients with or without K19 expression. K19-positive group had significantly lower overall survival rate than K19-negative group. Disease -free survival rate was not correlated with K19 expression. However, during an early phase, disease -free survival curve was lower in K19-positive group than in K19-negative group. The number of patients at risk at each time interval in K-19 positive group is showed beside each graph

Out of the total 136 HCC cases, 12 K19-positive HCC cases (8.8%) were examined in the present study (Fig. 1a). Results of an analysis of the relationship between K19 expression and various clinicopathological parameters are summarized in Table 3. K19-positive HCC predominantly occurred in young, female patients. K19-positive HCC was also associated with TNM stage, tumour differentiation (Fig. 1b), major vascular invasion, tumour-capsule formation as well as tumour necrosis. Early recurrence (within 6 months after surgery) frequently occurred in K19-positive cases. The percentages of extrahepatic recurrence were 41.7 and 10.5% in K19-positive and K19-negative cases, respectively (*p* = 0.002). Among the organs, metastasis to lung was most frequently observed in this study (*p* = 0.003). There was no significant difference between K19 expression and HBV or HCV infection. The non-HBV/non-HCV group and other pathological parameters such as microvascular invasion and fibrous stroma were not statistically correlated with K19 expression.

**Table 3:** Comparison of clinicopathologic features between K19-positive and K19- negative HCC (*n* = 136 cases)

Features	K19-positive group [*n* = 12 (8.8%)]	K19-negative group [*n* = 124 (91.2%)]	*P* value
Age (years, mean ± SD)	60.1 ± 16.4	69.7 ± 9.32	*0.002*
Gender (male:female) (male%)	5:7 (41.7)	98:26 (79.0)	*0.004*
Infection			
HBV (%)	4 (33.3)	29 (23.4)	0.443
HCV (%)	4 (33.3)	58 (46.8)	0.372
Non-HBV, non- HCV (%)	4 (33.3)	39 (31.5)	0.894
Cirrhosis (%)	3 (25.0)	40 (32.3)	0.606
Tumour size (mm, mean ± SD)	42.2 ± 33.3	37.0 ± 24.8	0.505
Multiple tumours (%)	2 (16.7)	14 (11.3)	0.581
TNM stage			*<0.001*
I-II (%)	4 (33.3)	63 (50.8)	
III-IV (%)	8 (66.6)	61 (49.2)	
Differentiation			*<0.001*
Well (%)	0 (0)	33 (26.6)	
Moderate (%)	7 (58.3)	84 (67.7)	
Poor (%)	5 (41.7)	7 (5.6)	
Major vascular invasion (%)	3 (25.0)	3 (2.4)	*<0.001*
Microvascular invasion (%)	10 (83.3)	68 (54.8)	0.063
Tumour-capsule formation (%)	5 (41.7)	98 (79.0)	*0.004*
Fibrous stroma (%)	5 (41.7)	41 (33.1)	0.575
Necrosis (%)	9 (75.0)	34 (27.4)	*<0.001*
Recurrence (%)	5 (41.7)	54 (43.5)	0.977
Early recurrence^a^ (%)	5 (41.7)	16 (12.9)	*0.005*
Extrahepatic recurrence (%)	5 (41.7)	13 (10.5)	*0.002*
Lung (%)	4 (33.3)	9 (7.3)	*0.003*
Bone (%)	1 (8.3)	2 (1.6)	0.130
Lymph nodes (%)	1 (8.3)	3 (2.4)	0.247
Adrenal gland (%)	0(0)	1 (0.8)	0.755

^a^Early recurrence within 6 months after surgery

Survival analysis demonstrated that patients with K19-positive HCC had significantly poorer overall survival than did patients with K19-negative HCC (*p* < 0.01) (Fig. 1c). In contrast, there was no significant difference in disease-free survival (*p* = 0.573) unless the data were analysed during an early phase (Fig. 1d). The multivariate analysis demonstrated that tumour size and necrosis were independent predictors of overall survival, but this was not the case with K19 expression (Additional file 2: Table S1).

### Induction of senescence and apoptosis by *K19* knockdown

**Fig. 2:**
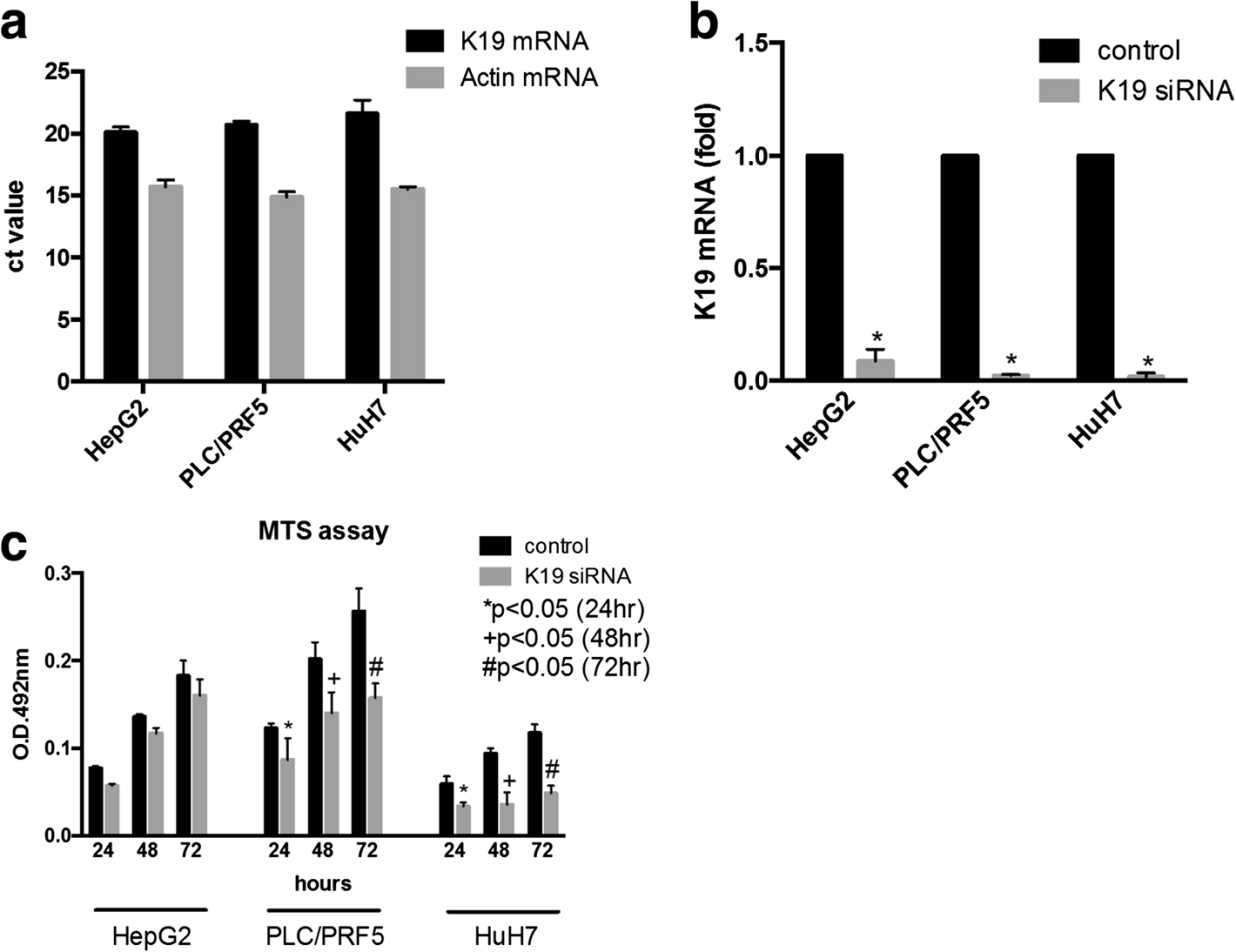
*K19* expression and cell growth in human HCC cell lines HepG2, PLC/PRF/5, and HuH-7. **a** Real-time PCR analysis revealed strong expression of *K19* in all cell lines. **b** *K19* expression in all cell lines was significantly reduced by transfection with siRNA for 72 -h. **c** Cell proliferation assay using methane thiosulfonate (MTS) reagent showed suppression of K19 inhibited tumour growth in PLC/PRF/5 and Huh-7 cells but not in HepG2 cells

**Fig. 3:**
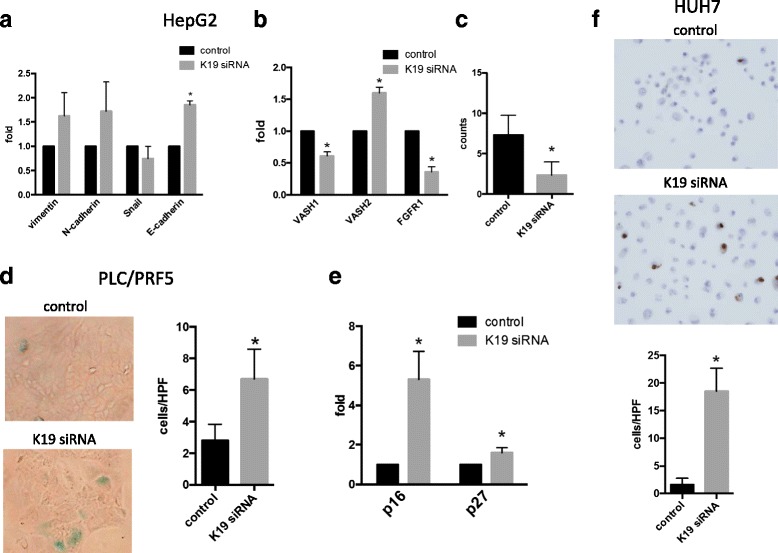
Cell invasion, senescence, apoptosis and angiogenesis in HCC cell lines. **a** E-cadherin mRNA expression increased, whereas vimentin, N-cadherin and snail mRNA expression was not significantly affected by *K19* knockdown in HepG2 cells. **b** The expression of angiogenesis -related genes *VASH1* and *FGFR1* decreased and that of *VASH2* increased following *K19* -knockdown in HepG2 cells. **c** Matrigel invasion assay showing inhibition of invasion capacity of HepG2 cells transfected with *K19* siRNA. **d** Analysis using SA-β-gal demonstrates significant induction of cell senescence in PLC/PRF5 cells following *K19* knockdown using siRNA transfection. **e** Senescence -related genes, *p16* and *p27*, upregulated by *K19* knockdown in PLC/PRF5 cells. **f** In liquid based cytology (LBC) of HuH-7 cells, the number of apoptotic cells increased following *K19* knockdown

In the current study, we used three human HCC cell lines, HepG2, PLC/PRF/5, and HuH-7, which express K19 strongly, as determined by real-time PCR (Fig. 2a). *K19* expression was successfully suppressed by transfection with *K19* siRNA, followed by 72-h incubation (Fig. 2b). As shown in Fig. 2c, cell growth was significantly suppressed by *K19* knockdown in PLC/PRF/5 and HuH-7 cells but not in HepG2 cells. When PLC/PRF/5 cells were transfected with *K19* siRNA, senescence was induced, as assessed by SA-β-gal assay (Fig. 3d). Furthermore, *K19* silencing upregulated mRNA levels of senescence-related genes such as *p16* and *p27* in PLC/PRF/5 cells (Fig. 3e). In LBC of HuH-7 cells, the number of apoptotic cells increased following *K19* knockdown (Fig. 3f). Considered together, it appears that *K19* knockdown induced apoptosis in HuH-7 cells and senescence in PLC/PRF/5 cells through the upregulation of *p16* and *p27* genes.

### *K19* knockdown increased *E-cadherin* gene expression, and inhibited cancer invasion and angiogenesis

**Fig. 4:**
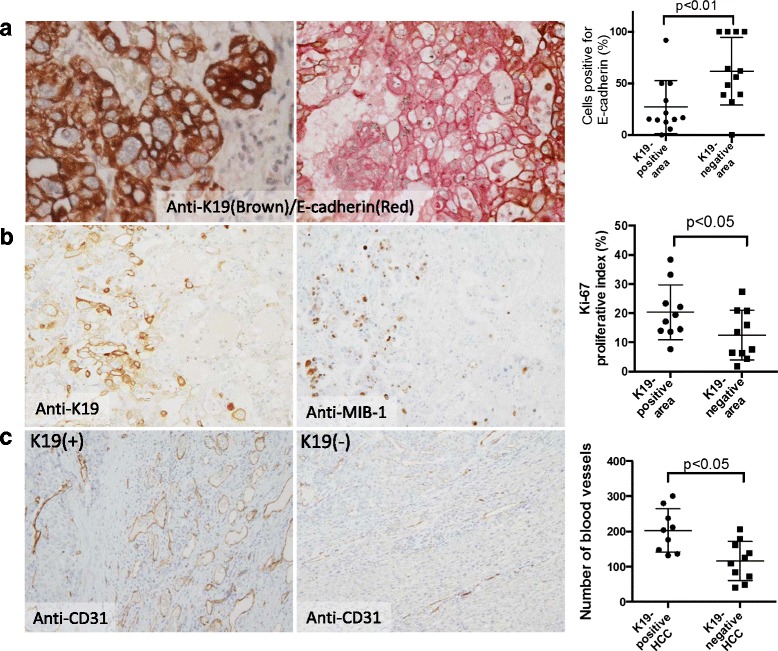
Immunohistochemical analysis of human HCC specimens. **a** Double immunostaining for K19 (cytoplasm, brown) and E-cadherin (membrane, red) showed that the percentage of cells positive for E-cadherin in K19-positive areas was lower than that in K19-negative areas of K19-positive HCC specimens. **b** Mirror image analysis of K19 and Ki-67 indicated Ki-67 proliferative index was higher in K19-positive areas than in K19-negative areas of K19-positive HCC specimens. **c** K19-positive HCC specimens had more CD31-positive blood vessels around cancer foci than did K19-negative HCC specimens. Original magnifications: ×400 (a, left), ×200 (a, right), ×100 **b, c**

As stated above, cell growth was not significantly affected by *K19* siRNA transfection of HepG2 cells. In the light of this result, we examined the effect of *K19* knockdown on cancer invasion and angiogenesis. Figure 3a and c indicate that E-cadherin gene expression and matrigel invasion capacity of HepG2 cells increased and decreased, respectively, following *K19* silencing. This suggests that *K19* could enhance cancer invasion through decreased E-cadherin gene expression in HCC cells. Gene expression levels of Vimentin, N-cadherin, and snail were not affected by *K19* knockdown (Fig. 3a). The expression of the angiogenesis-related genes *VASH1* and *FGFR1* decreased, while that of *VASH2* increased following *K19* knockdown in HepG2 cells (Fig. 3b). However, immunohistochemical analysis of HCC specimens indicated that VASH1 is strongly expressed not only in K19-positive HCC cells, but also in K19-negative HCC cells. VASH1 expression in HCC was not statistically correlated with K19 expression. Finally, we examined the E-cadherin expression and the HCC proliferative activity in both K19-positive and K19-negative areas using human K19-positive HCC specimens. Double immunohistochemical staining clearly showed that the percentages of cells positive for E-cadherin were 27.2% in K19-positive areas and 61.7% in K19-negative areas (*p* < 0.01) (Fig. 4a). In contrast, the Ki-67 proliferative index was higher in the K19-positive areas than in K19-negative areas (Fig. 4b). The Ki-67 proliferative index in the K19-negative area was similar to that observed in the K19-negative HCC specimens. Furthermore, the number of blood vessels around cancer foci was significantly higher in K19-positive HCC specimens than in K19-negative HCC specimens (Fig. 4c). These pathological data were thus in agreement with the results from the in vitro experiments.

## Discussion

In the current study, we demonstrated that K19 promoted HCC invasion, proliferation, and angiogenesis, using in vitro experiments and immunohistochemistry. Survival analysis revealed that patients with K19-positive HCC had significantly poorer overall survival than did patients with K19-negative HCC, although K19 expression was not an independent predictor in the multivariate analysis for overall survival. In previous reports, K19-positive HCC demonstrated higher invasiveness, greater metastatic potential, and poorer prognosis than did conventional HCC. Moreover, K19-positive HCC specimens examined had greater vessel invasion, poor differentiation, greater infiltrative growth, and more extrahepatic metastasis than did K19-negative HCC specimens [5, 8]. Although these pathological characteristics are well documented, the biological mechanisms involved in the aggressive behaviour of K19-positive HCC remain unclear.

The keratins, which are intermediate filament proteins, play several important roles within the cell. For instance, they maintain the mechanical stability and integrity of epithelial cells, as well as participate in several intracellular signalling pathways involved in coping with cell stress [18]. K19 is the smallest keratin, as it lacks the non-α-helical tail domain, which is typical of all other keratins [19]. This protein also appears functionally dispensable because *K19* knockout mice were viable, fertile, and appear normal [20]. In the present study, K19 enhanced cancer invasion by decreasing E-cadherin expression, and promoted cell survival by suppressing the induction of senescence and apoptosis in HCC cells. However, the effects of K19 were not the same across all three cell lines used in this study, which may be due to differences in the roles of K19, such as in cellular differentiation, in the biological subtypes.

Ozturk et al. [21] reported that HCC cells bypass the senescence barrier by inactivating major senescence-related genes such as *p53*, *p16* ^*INK4a*^ and *p15* ^*INK4*^. *p16* is well known to induce cell quiescence, which is tightly associated with cell differentiation. Thus, *K19* could inhibit HCC cell differentiation by regulating *p16*. Apoptosis was induced by *K19* knockdown in vitro; however, the TUNEL assay did not indicate a significant difference in apoptosis induction between K19-positive and K19-negative HCC areas. The percentage of Ki-67-positive cells was statistically higher in K19-positive HCC areas than in K19-negative areas. Considered together with the in vitro data, K19 appears to promote HCC cell proliferation, and its suppression effectively inhibits tumour growth via induction of cytotoxicity.

Recently, Govaere et al. [11] reported for the first time that *K19* knockdown in HCC cell line resulted in reduced invasive ability. We found that *K19* promotes cancer invasion in HepG2 cells through the downregulation of E-cadherin gene expression. Gene expression of snail, N-cadherin, and vimentin was not affected by *K19* knockdown. Kim et al. [8] reported that K19-positive HCC was not associated with loss of E-cadherin expression in tissue microarray study. Using double immunostaining of K19 and E-cadherin, we clearly showed that the percentage of cells positive for E-cadherin in K19-positive areas was lower than that in K19-negative areas of K19-positive HCC specimens. Decreased E-cadherin expression was also shown in invasive lobular carcinoma of the breast. In this case, E-cadherin downregulation is caused by promoter methylation, mutations, or loss of heterozygosity (LOH) [22]. The mechanism underlying the decrease in E-cadherin expression in K19-positive HCC should be one of the goals of future investigations.

We showed here that *K19* upregulated *FGFR1* and *VASH1* and downregulated *VASH2* in HCC cells. Moreover, immunochemical analysis showed increased blood vessels in K19-positive HCC. FGFR1 is a receptor tyrosine kinase that activates endothelial-cell proliferation and migration [23]. Thus, it is expected that FGFR1 could be a useful therapeutic target [24]. Recent investigations focused on the roles of VASH1 and VASH2 as new regulators in angiogenesis. VASH1 is a negative feedback regulator of angiogenesis, whereas VASH2 promotes angiogenesis [25, 26]. Several studies have shown that VASH1 expression in HCC is associated with vascular invasion and poor prognosis [27, 28]. VASH may have different functions in HCC, and it is necessary to analyse its organ-specific functions. Immunohistochemical analysis of HCC specimens indicated that VASH1 is strongly expressed not only in K19-positive but also in K19-negative HCC cells. We have not excluded the possibility that K19 might control other signals of VASH1-dependent angiogenesis in HCCs. *K19* may enhance tumour angiogenesis by regulating *FGFR1*, *VASH1*, and *VASH2* in HCC. Yoneda et al. [3] reported that epidermal growth factor (EGF) promoted growth and invasiveness in HCC, which was accompanied by increased K19 expression. EGF might be associated with tumour growth and invasion as a molecule downstream of K19.

## Conclusions

Our findings clearly indicate that K19 has a direct role in promoting HCC cell survival and invasion by inhibiting senescence and apoptosis and downregulating E-cadherin gene expression, respectively. In addition, *K19* enhanced angiogenesis by affecting the expression of angiogenesis-related genes such as *VASH1*, *VASH2*, and *FGFR1.* Thus, K19 directly promotes cancer cell survival, invasion, and angiogenesis. K19 could be a new target molecule for the development of therapies against K19-positive HCC.

## Additional files

Additional file 1: Figure S1. Positive and negative controls for immunohistochemistry. (PPTX 1056 kb)
Additional file 2: Table S1. Multivariate analysis for overall survival. (DOCX 13 kb)

## Abbreviations

EMT: Epithelial-mesenchymal transition
FGFR: Fibroblast growth factor
HBsAg: Hepatitis B virus surface antigen
HCC: Hepatocellular carcinoma
HCVAb: Hepatitis C virus antibody
K: Keratin
LBC: Liquid based cytology
TUNEL: Terminal deoxynucleotidyl transferase-mediated deoxyuridine triphosphate-biotin nick end labeling
VASH: Vasohibin
