# Supplementary material for: Keratin 19 as a key molecule in progression of human hepatocellular carcinomas through invasion and angiogenesis
Source: BMC Cancer. 2016 Nov 18;16:903. doi: 10.1186/s12885-016-2949-y (PMC5116168; doi:10.1186/s12885-016-2949-y)
Supplement: Additional file 1: Figure S1. — Positive and negative controls for immunohistochemistry. (PPTX 1056 kb) [file 12885_2016_2949_MOESM1_ESM.pptx]

## Slide 1
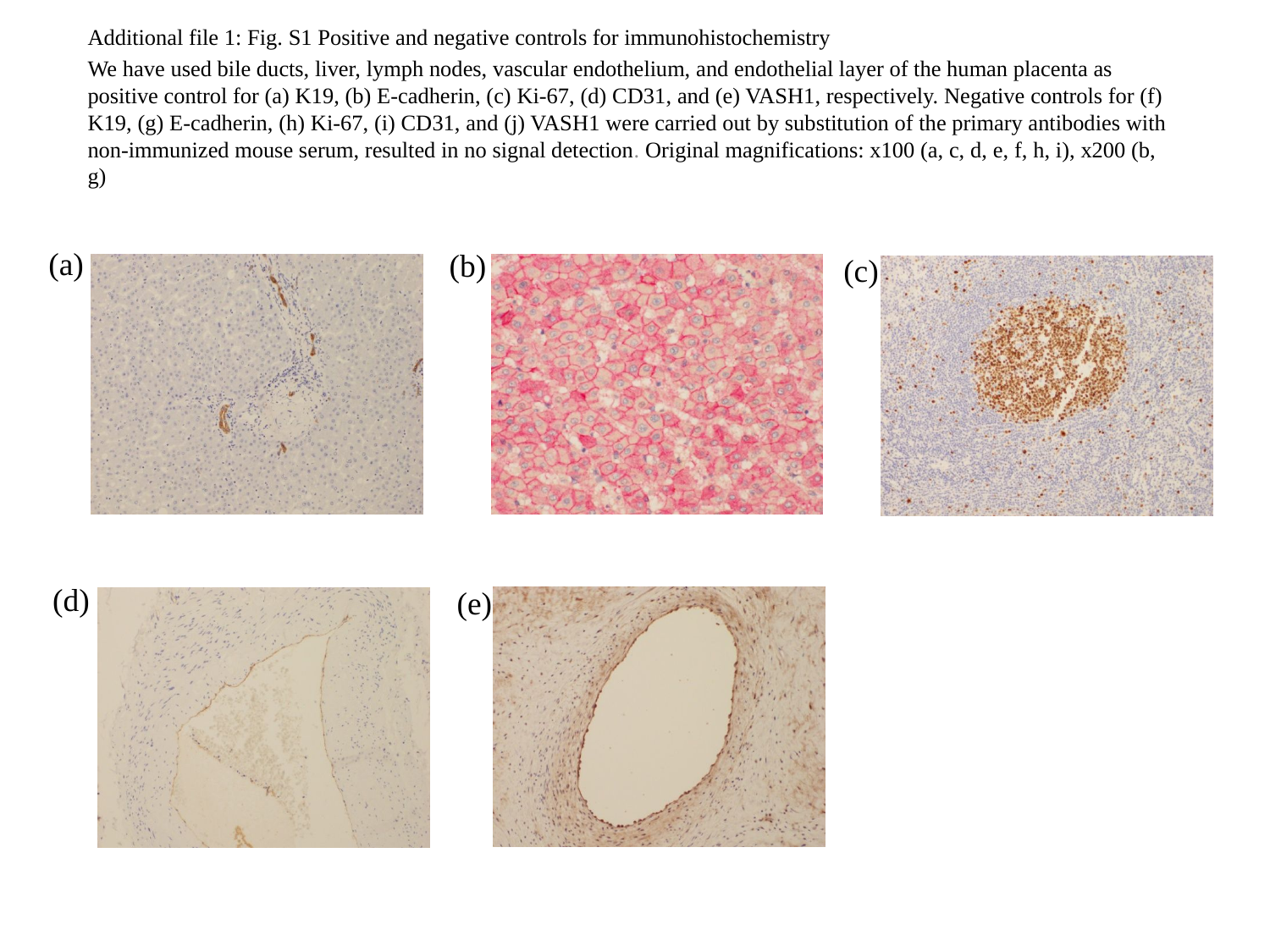

Additional file 1: Fig. S1 Positive and negative controls for immunohistochemistry
We have used bile ducts, liver, lymph nodes, vascular endothelium, and endothelial layer of the human placenta as positive control for (a) K19, (b) E-cadherin, (c) Ki-67, (d) CD31, and (e) VASH1, respectively. Negative controls for (f) K19, (g) E-cadherin, (h) Ki-67, (i) CD31, and (j) VASH1 were carried out by substitution of the primary antibodies with non-immunized mouse serum, resulted in no signal detection. Original magnifications: x100 (a, c, d, e, f, h, i), x200 (b, g)
(a)
(b)
(c)
(d)
(e)

## Slide 2
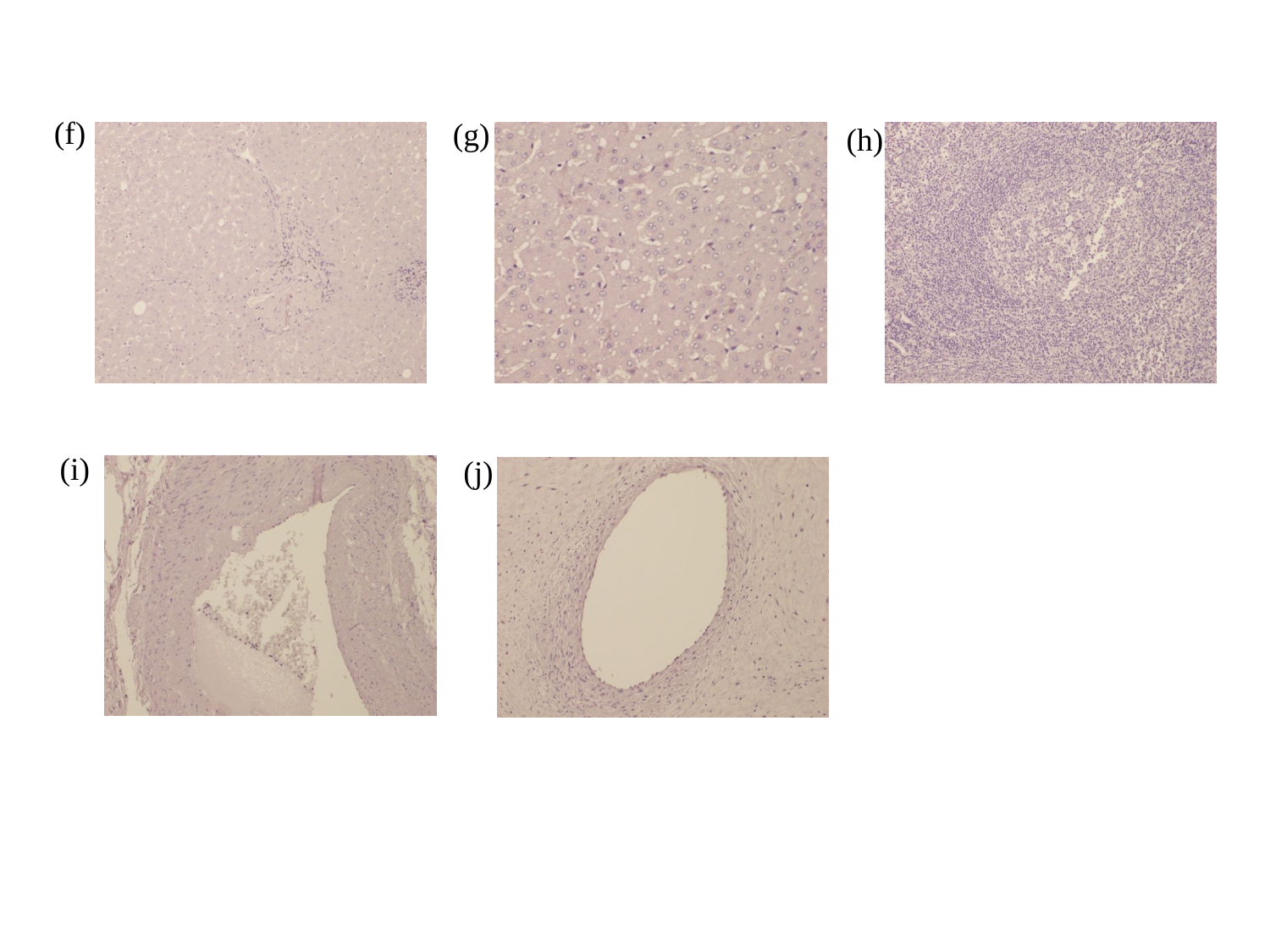

(f)
(g)
(h)
(i)
(j)
