# Supplementary material for: Keratin 19 as a key molecule in progression of human hepatocellular carcinomas through invasion and angiogenesis
Source: BMC Cancer. 2016 Nov 18;16:903. doi: 10.1186/s12885-016-2949-y (PMC5116168; doi:10.1186/s12885-016-2949-y)
Supplement: Additional file 2: Table S1. — Multivariate analysis for overall survival. (DOCX 13 kb) [file 12885_2016_2949_MOESM2_ESM.docx]

Additional file 2: Table S1: Multivariate analysis for overall survival was performed using a Cox regression model with forward stepwise selection. The multivariate analysis demonstrated that tumour size and necrosis were independent predictors of overall survival, but this was not the case with K19 expression.

| Variable | Multivariate risk ratio | *P* value |
| --- | --- | --- |
| Keratin 19 expression | 3.18(0.17-65.14) | 0.4307 |
| Tumor size | 1.04(1.01-1.08) | **0.003** |
| Necrosis | 6.76(1.32-49.83) | **0.0209** |
